# Platelet Activation Is Triggered by Factors Secreted by Senescent Endothelial HMEC-1 Cells In Vitro

**DOI:** 10.3390/ijms21093287

**Published:** 2020-05-06

**Authors:** Whitney Venturini, Alexandra Olate-Briones, Claudio Valenzuela, Diego Méndez, Eduardo Fuentes, Angel Cayo, Daniel Mancilla, Raul Segovia, Nelson E. Brown, Rodrigo Moore-Carrasco

**Affiliations:** 1 Center for Medical Research, University of Talca Medical School, Talca 3460000, Chile; whitneyventurini@gmail.com (W.V.); amolateb@gmail.com (A.O.-B.); cvalenzuela@utalca.cl (C.V.); acayo@utalca.cl (A.C.); daniel.enrique.mancilla@gmail.com (D.M.); ralemilio@gmail.com (R.S.); 2 Faculty of Health Sciences, University of Talca, Talca 3460000, Chile; dmendez12@alumnos.utalca.cl (D.M.); edfuentes@utalca.cl (E.F.); 3 Facultad de Ciencias Biológicas, Pontificia Universidad Católica de Chile, Santiago 7500000, Chile; 4 Núcleo Científico Multidisciplinario, Universidad de Talca, Talca 3460000, Chile; 5 Thrombosis Research Center, Medical Technology School, Department of Clinical Biochemistry and Immunohaematology, Faculty of Health Sciences, University of Talca, Talca 3460000 Chile; 6 Programa de Investigación Asociativa en Cáncer Gástrico (PIA-CG), Talca 3460000, Chile

**Keywords:** platelets, cellular senescence, doxorubicin, endothelial cells, SASP

## Abstract

Aging is one of the main risk factors for the development of chronic diseases, with both the vascular endothelium and platelets becoming functionally altered. Cellular senescence is a form of permanent cell cycle arrest initially described in primary cells propagated in vitro, although it can also be induced by anticancer drugs and other stressful stimuli. Attesting for the complexity of the senescent phenotype, senescent cells synthesize and secrete a wide variety of bioactive molecules. This “senescence-associated secretory phenotype” (SASP) endows senescent cells with the ability to modify the tissue microenvironment in ways that may be relevant to the development of various physiological and pathological processes. So far, however, the direct role of factors secreted by senescent endothelial cells on platelet function remains unknown. In the present work, we explore the effects of SASP factors derived from senescent endothelial cells on platelet function. To this end, we took advantage of a model in which immortalized endothelial cells (HMEC-1) were induced to senesce following exposure to doxorubicin, a chemotherapeutic drug widely used in the clinic. Our results indicate that (1) low concentrations of doxorubicin induce senescence in HMEC-1 cells; (2) senescent HMEC-1 cells upregulate the expression of selected components of the SASP and (3) the media conditioned by senescent endothelial cells are capable of inducing platelet activation and aggregation. These results suggest that factors secreted by senescent endothelial cells in vivo could have a relevant role in the platelet activation observed in the elderly or in patients undergoing therapeutic stress.

## 1. Introduction

Aging has been traditionally considered an independent risk factor for the development of chronic conditions, most typically cardiovascular and neoplastic diseases [1,2]. Several interrelated “hallmarks of aging”, defined at the cellular and molecular levels, are thought to underlie organismal aging. These include genomic instability, telomere attrition, mitochondrial dysfunction, cellular senescence, stem cell exhaustion, and changes in intercellular communication [3].

The involvement of aging in the onset and progression of cardiovascular diseases has been widely documented [4,5]. In this context, aging has been linked to endothelial dysfunction, arterial rigidity and remodeling, dysfunctional angiogenesis and atherosclerosis [6]. Aging also affects various components of the hemostatic system [7]. Thus, age-dependent increases in coagulation factors, fibrinogen and von Willebrand factor in the serum [8], as well as activation of the anti-fibrinolytic system [8,9], have been described. Importantly, platelets from older individuals display greater aggregation responses to ADP and collagen when compared to platelets derived from younger individuals [10]. Moreover, indirect platelet-activation parameters, such as plasma levels of thromboxane A and PF4, are often elevated in older individuals [11,12]. Though the cellular and molecular bases of these age-related changes are complex, cellular senescence has emerged as an important contributor to both age-related loss of organ function and the development of cardiovascular and other chronic diseases [4].

Cellular senescence is a unique form of cell cycle arrest characterized by specific changes in morphology, gene expression and function [13]. First described in human primary cells subjected to long-term culture [14], a similar phenotype can be triggered prematurely in response to various forms of stress, including genotoxic, oxidative, oncogenic and therapeutic stress [15]. Indeed, several anticancer drugs are capable of inducing cellular senescence in cancer cells, including conventional chemotherapeutic drugs (i.e., Doxorubicin) [16] and more recently developed CDK4/6 inhibitors [17,18]. Importantly, cellular senescence has also been described in developmental contexts, in the absence of stressful stimuli [19,20,21], and senescent cells accumulate in aging tissues and sites of tissue damage [19,20]. 

Despite being unable to proliferate, senescent cells are capable of synthesizing and secreting a complex mix of growth factors, proteases, cytokines and components of the extracellular matrix [13,22]. This feature, referred to as the senescence-associated secretory phenotype (SASP), raises the possibility that many of the effects of senescent cells in tissues might be essentially non-cell-autonomous. Interestingly, proinflammatory molecules are among the most highly conserved SASP components [13,23,24,25], suggesting a link between the accumulation of senescent cells and age-related chronic inflammation [15]. Nonetheless, the in vivo consequences of factors secreted by senescent cells are often difficult to predict, probably due to cell-type- and stimulus-dependent variations in SASP composition. Thus, while some SASP components can propagate or reinforce cellular senescence [22,24,26,27], other components promote proliferation, migration or invasion of premalignant and malignant cells [23,25,28,29]. These complexities are particularly evident in the interaction between senescent cells and immune cells: while some SASP components may recruit immune cells with anti-cancer properties (e.g., NK cells, macrophages, and Th1 cells) [30,31], others generate immunosuppressive microenvironments that promote cancer progression [32].

So far, the role of senescent endothelial cells and their secreted factors as modulators of platelet activity has only been suggested as a theoretical possibility, based on indirect observations [33]. Interestingly, interleukin 6 (IL-6), a proinflammatory cytokine and one of the most prominent factors secreted by senescent cells [22], can directly activate platelets [34]. As levels of IL-6 are also strongly correlated with aging [35], these observations suggest that high levels of IL-6 (and other proinflammatory factors) might, at least in part, reflect high rates of secretion of this cytokine by senescent cells in the context of aging or age-related inflammation.

Here, we show that doxorubicin-induced senescent endothelial cells upregulate the expression of various SASP components (IL-6, IL1-β, IL-8). More importantly, media conditioned by these senescent endothelial cells are capable of inducing platelet activation and aggregation. These results suggest that factors secreted by senescent endothelial cells in vivo could have a relevant role in the platelet activation observed in the elderly, or as a consequence of cellular senescence induced by therapeutic stress.

## 2. Results

### 2.1. Doxorubicin Induces Senescence in HMEC-1 Cells

**Figure 1:**
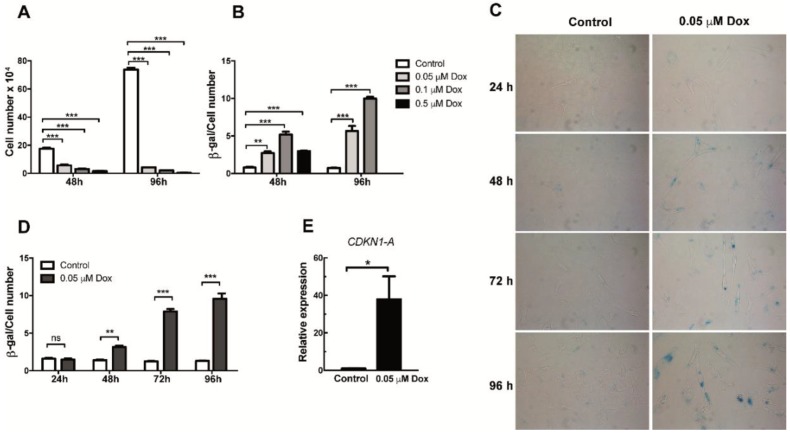
Analysis of proliferation and senescence in doxorubicin-treated HMEC-1 cells. (**A**) Number of HMEC-1 cells treated with three different concentrations of doxorubicin for 48 and 96 h. (**B**) Senescence-associated (SA)-β Galactosidase (SA-β-Gal) activity in doxorubicin (Dox)- and vehicle-treated (control) HMEC-1. Quantification was based on color intensity corrected by the number of cells. (**C**) Representative images of SA-β-Gal staining in HMEC-1 cells following treatment with 0.05 μM of doxorubicin for 24, 48, 72 and 96 h. (**D**) Quantification of SA-β-Gal activity in HMEC-1 cell treated with 0.05 μM of doxorubicin for 24, 48, 72 and 96 h. (**E**) Expression analysis of *CDKN1* (encoding p21^CIP1/KIP1^) RNA levels in cells treated with 0.05 μM of doxorubicin. Error bars indicate mean ± SD of *n* = 3 (NS = no significant; * *p* < 0.05; ** *p* < 0.01; *** *p* < 0.001; *t*-student test).

In order to generate senescent endothelial cells in vitro, HMEC-1 cells were initially cultured for 48 or 96 h in the presence of increasing concentrations (0.05, 0.1 and 0.5 µM) of doxorubicin. As shown in Figure 1A, a reduction in proliferation was evident when cells were exposed for 48 h to all three concentrations of doxorubicin. This reduced proliferation was further exacerbated when a 96 h-treatment schedule was tested (Figure 1A). After confirming doxorubicin’s effects on proliferation, we proceeded to determine if this effect was associated with induction of cellular senescence. To this end, the senescence-associated β-galactosidase (SA-β-Gal) assay, a widely used senescence biomarker, was performed on control and doxorubicin-treated cells. After quantifying SA-β-Gal positive staining and normalizing it by the total amount of cells in a microscopic field, we observed an increase in the proportion of senescent cells among those cells that were exposed to increasing doses of doxorubicin for 48 or 96 h (Figure 1B–D). The induction of senescence was evident even with the lowest dose of doxorubicin (0.05 μM) (Figure 1B–D). In contrast, when higher doses were used (0.5 μM), cell viability was compromised, particularly in the setting of 96-h treatment (Figure 1B). Based on these results, a 0.05-μM concentration of doxorubicin was used to induce senescence in HMEC-1 cells in the experiments described below. Indeed, when cells were treated for 24, 48, 72 and 96 h with 0.05 μM of doxorubicin, both the staining intensity and the proportion of stained cells increased with time, with a plateau reached after 72 and 96 h of treatment (Figure 1D). Of note, SA-β-Gal positive cells showed a characteristic increase in the size of senescent cells (Figure 1C). Importantly, senescence only became evident after 48 h of exposure to doxorubicin (Figure 1D). As an additional way to corroborate senescence induction, we also tested the mRNA expression of the cyclin-dependent kinase inhibitor *CDKN1A* (also known as p21^CIP1/KIP1^) 72 h after exposure to doxorubicin. As shown in Figure 1E, an increase in the mRNA levels of this senescence marker can be observed in doxorubicin-treated HMEC-1 cells. From these results, 72 h was selected as the time in which the expression of SASP factors could be detected.

### 2.2. Senescent Endothelial Cells Express SASP Factors

**Figure 2:**
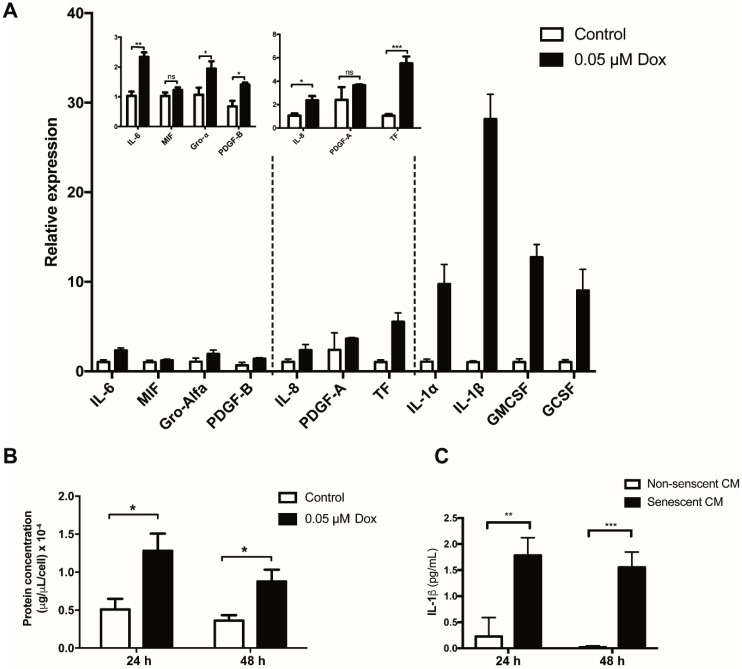
Expression of selected senescent-associated secreted factors in senescent HMEC-1 cells. (**A**) qRT-PCR-based expression analyses of selected SASP factors in doxorubicin (Dox)-treated HMEC-1 cells. (**B**) Total protein concentration, corrected by cell number, in conditioned media generated by senescent and non-senescent (control) cells. (**C**) Protein levels of IL-1β present in conditioned media from senescent and no-senescent HMEC-1 cells were determined by ELISA. Error bars indicate mean ± SD of *n* = 3 (NS = not significant; * *p* < 0.05; ** *p* < 0.01; *** *p* < 0.001; *t*-student test).

One of the most important pathophysiological features of senescent cells is their ability to produce and secrete a wide variety of soluble and insoluble factors. Therefore, one of our aims was to identify changes in the expression of SASP components in senescent HMEC-1 cells. To this end, a list of factors secreted by senescent cells was compiled based on their ability to directly or indirectly affect platelet aggregation. Expression of these factors was determined by quantitative real-time PCR (qRT-PCR). As shown in Figure 2A, treating HMEC-1 cells with 0.05 µM doxorubicin for 72 h led to an increase in the expression of all analyzed factors, with the exception of PDGF-A and MIF (macrophage migration inhibitory factor). Of note, the expression of interleukin 1 beta (IL-1β) was particularly enhanced in senescent HMEC-1 cells (Figure 2A). Overall, senescent cells secreted greater amounts of total protein per cell, when compared to cells in active proliferation (non-senescent cells): 24 or 48 h after ending doxorubicin treatment, senescent endothelial cells secrete 2.4 or 2.6 times more protein than their non-senescent counterparts, respectively (Figure 2B). Accordingly, the protein levels of IL-1β in media conditioned by senescent HMEC-1 cells were much higher than the levels detectable in media conditioned by non-senescent HMEC-1 cells (Figure 2C).

### 2.3. Media Conditioned by Senescent Endothelial Cells Induce Platelet Adhesion and Aggregation

**Figure 3:**
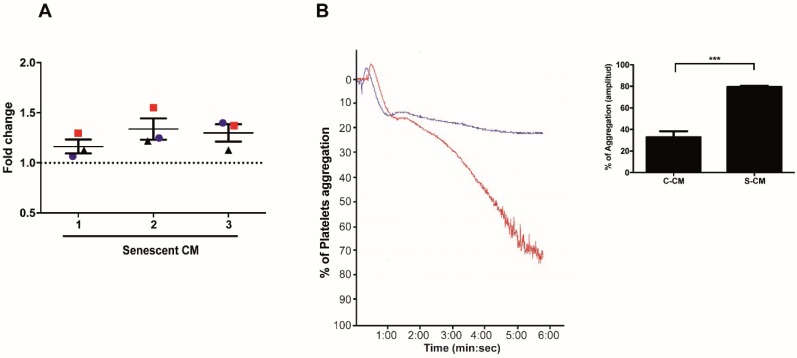
Effects of media conditioned by senescent cells on platelet aggregation and adhesion. (**A**) Adhesion (assessed under static conditions) of platelets exposed to media conditioned by senescent and non-senescent HMEC-1 cells. The graph represents the fold change increase in platelet adhesion in the presence of media conditioned by senescent cells; the basal, discontinuous, line represents the basal adhesion of platelets exposed to media conditioned by non-senescent cells. Three donors were tested (red squares, blue circles and black triangles) with conditioned media. (**B**) Representative images of time-course recordings of platelet aggregation assays carried out with platelet-enriched plasma incubated with conditioned media collected from senescent (blue curve) and non-senescent (red curve). The percentage of maximum platelet aggregation for three independent experiments is shown (*n* = 3; *** *p* < 0.001; *t*-student test). Dox: doxorubicin. C-CM: control conditioned media; S-CM: senescent conditioned media.

Little is known about the effects of factors secreted by senescent endothelial cells on platelet function. In order to study these effects, we first assessed the capacity of media conditioned by senescent and non-senescent HMEC-1 cells to promote platelet adhesion and aggregation. Platelet adhesion, assessed under static conditions, revealed a higher capacity of senescent conditioned media to promote the adhesion of washed platelets on collagen-coated 96 well plates (Figure 3A). Similarly, incubation of platelet-rich plasma (PRP) with media previously conditioned by senescent (red curve) or non-senescent (blue curve) HMEC-1 cells led to 70% and 20% of platelet aggregation, respectively (Figure 3B). A quantification of the values of aggregation (amplitude) indicated that media conditioned by senescent (doxorubicin-treated) cells were able to induce more prominent platelet aggregation than media collected from non-senescent cells (the percentage of platelet aggregation in platelets previously incubated with senescent conditioned media increased by 47%) (Figure 3B, graph).

### 2.4. Platelet Activation Is Promoted by Factors Secreted by Senescent HMEC-1 Cells

**Figure 4:**
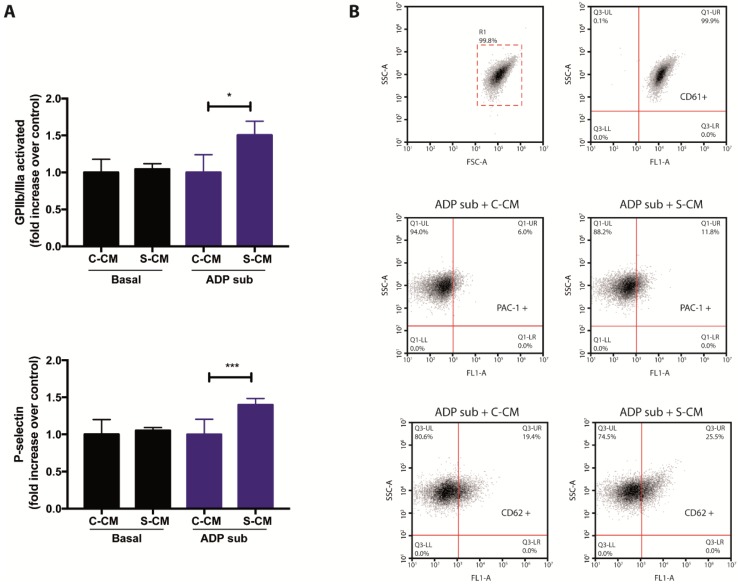
Effects of media conditioned by senescent cells on platelet activation. (**A**) The presence of activated GPIIb/IIIa and P-selectin on the surface of platelets, previously exposed to media conditioned by senescent and non-senescent HMEC-1 cells, was determined by flow cytometric analyses. Platelets under basal and sub-aggregating (ADP sub) conditions were tested. (**B**) Representative dot plots for the detection of CD61 (platelets; top), PAC-1 (activated GPIIb/IIIa; middle) and CD62 (P-selectin; bottom) on human platelets. ADP sub: 1.3–2.0 μM ADP; C-CM: Control conditioned medium; S-CM: Senescent conditioned medium; SSC: side scatter; FSC: forward scatter. The graph depicts the mean ± SD of *n* = 4 (PAC-1) and *n* = 7 (CD62) experiments. * *p* < 0.05 and *** *p* < 0.001 analyzed by Student’s *t*-test.

Finally, in order to complement the results shown in Figure 2 and Figure 3, we assessed flow-cytometry-based signs of activation in platelets previously exposed to media conditioned by senescent endothelial cells. To this end, we took advantage of antibodies recognizing the active conformation of the αIIbβ3 integrin receptor (also known as glycoprotein GPIIb/IIIa), as well as the presence of P-selectin, on the surface of platelets. Importantly, media conditioned by senescent cells were tested both in the absence or presence sub-aggregating concentrations of ADP. As shown in Figure 4 (panels A and B), the addition of senescent conditioned media was not able to induce platelet activation (cytometry-based detection of αIIbβ3 and P-selectin) under basal conditions. However, under sub-aggregating conditions, the addition of media conditioned by senescent cells led to cytometric signs of platelet activation (Figure 4, panels A and B), confirming the presence of platelet-activating factors in the secretome of senescent HMEC-1 cells. Altogether, our results suggest a novel and pathophysiologically relevant interaction between senescent endothelial cells and platelets.

## 3. Discussion

**Figure 5:**
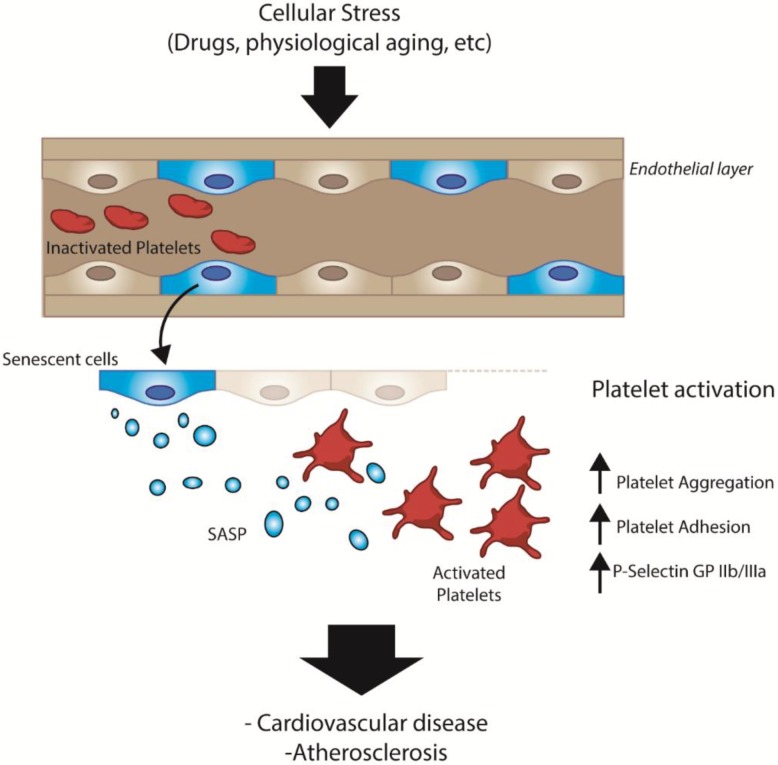
General scheme depicting the SASP-dependent interactions between senescent endothelial cells and platelets in vivo.

In this work, we present indirect evidence suggesting that senescent endothelial cells, derived under physiological or pathological conditions, can activate platelets via paracrine mechanisms (Figure 5). Activation of platelets, in turn, can lead to an array of disorders marked by thrombus formation. Several studies have shown that senescent cells accumulate in tissues as a function of age [36,37], contributing to the development of chronic diseases, including thrombotic events, myocardial infarction and hypertension [38]. In this context, our work provides additional evidence linking aging and the development of cardiovascular diseases [4]. While several hallmarks of aging have been proposed [3], our work also highlights the relevance of factors specifically secreted by senescent endothelial cells as mediators of platelet activation [4,39].

Previous observations have revealed several age-dependent changes in endothelial cells, including a reduced proliferative potential and reduced bioavailability of nitric oxide and production of prostaglandins [38]. In addition, senescent endothelial cells increase the production and secretion of inflammatory cytokines (e.g., IL-6 and IL-8) and adhesion molecules (e.g., ICAM-1) [6,40]. However, how factors secreted by senescent endothelial cells impinge on platelets remains unknown. Of note, the effects of aging on components of the hemostatic system, including platelets, are well known [8]. In addition to their role in the last steps of the atherothrombotic process [41,42,43], platelets also serve as a bridge that allows interaction between endothelial cells and monocytes. This interaction precedes the transmigration of monocytes to the intima and their subsequent transformation into macrophages and foam cells [42].

In order to study the paracrine effects of senescent endothelial cells on platelets, we first generated an in vitro model of drug-induced senescence. In this model, immortalized endothelial cells (HMEC-1), originally derived from human dermal microvasculature [44], were induced to senesce after being exposed to low doses of the chemotherapeutic drug doxorubicin. Reduced doses of this drug were chosen, among other alternatives, based on its reduced cytotoxic effects. doxorubicin exerts its anti-proliferative action through several mechanisms, including induction of cellular senescence [45]. There is evidence that doxorubicin-induced senescence is dependent on the activation of p53 [46] and p21 [47].

Importantly, ROS production has been suggested as one of the main mechanisms through which doxorubicin induces senescence in endothelial cells in vitro [48]. In this context, ROS appear to activate the senescence program by regulating p38 and JNK mitogen-activated protein kinases and triggering p16^INK4a^-dependent signaling [49]. On the other hand, a significant increase in ROS, accompanied with mitochondrial dysfunction and apoptosis, has been reported in endothelial cells of mice treated with doxorubicin [50]. So far, however, there have been no reports describing the effect of low doses of doxorubicin on HMEC-1 cells.

It is important to emphasize that, while not directly extrapolatable to the in vivo interactions between senescent endothelial cells and platelets, our in vitro model is nevertheless useful as a first step to study these interactions in the context of other pathophysiologic conditions. For example, there is considerable evidence that chemotherapy per se increases the risk of venous thrombosis [51,52,53], partly because of its effects on vascular endothelial cells, platelets and monocytes [51,54]. Thus, our model could help explain the role of drug-induced endothelial senescence in the generation of thrombotic events in patients undergoing chemotherapy. In line with this, it has been shown that doxorubicin increases the risk of venous thrombosis [55], an effect that could be mediated by an increase in platelet activity [56]. Whether platelet activation in these settings is due to the effects of senescent endothelial cells, doxorubicin or both, remains unknown. Of note, while high concentrations of doxorubicin (ranging from 30 to 250 µM) can directly induce platelet cytotoxicity and procoagulant activities, concentrations below 30 µM have no effect on platelet function [56,57,58].

While platelet-activating factors secreted by senescent endothelial cells await a more comprehensive characterization, our expression analyses of selected factors (selected according to their previously described roles in atherosclerosis and platelet function) revealed several candidates, a subset of which are currently being tested in our laboratory either individually or in combination. Thus, the expression of IL-1β in senescent HMEC-1 cells increased more than 28 times at the mRNA level and more than 10 times at the protein level, compared to non-senescent control cells (Figure 3). IL-1β is a known mediator of endothelial activation in the context of atherosclerosis [59,60]. IL-1β induces the secretion of IL-6, IL-8 and MCP-1 (monocyte chemo-attractant protein 1) [60,61], and also induces the endothelial expression of ICAM-1 (intercellular adhesion molecule-1) and αvβ3, two important mediators of neutrophil and monocyte adhesion to the endothelium under inflammatory conditions [61]. Accordingly, lack of IL-1β decreases the severity of atherosclerosis in mice [62]. Besides IL-1β, the expression of other proinflammatory factors, known to be involved in atherogenesis, was increased in senescent HMEC-1 cells. Relevant among these are IL-8, a chemokine involved in the “rolling” of monocytes on the endothelial surface during the early stages of atherogenesis [63], GRO-α, a chemokine capable of activating and retaining monocytes in the atherosclerotic lesion and inducing neutrophil chemotaxis [64], and IL-6, a pleiotropic cytokine that has been shown to increase the formation of atherosclerotic lesions in mice [65,66] and induce the expression of adhesion molecules in endothelial cells [67]. Interestingly, IL-6 levels are elevated in the sera of older adults [35,68]. It is important to mention that the receptors for IL-1β and IL-6 are also expressed on the surface of platelets [69,70,71]. While IL-8 receptors have not been identified in platelets, recent studies have shown that IL-8 is capable of inducing platelet activation [71]. Other SASP components that have been linked to atherosclerosis, and whose expressions were increased in our model of senescence, include the granulocyte-colony stimulating factor (G-CSF), the granulocyte macrophage-colony stimulating factor (GM-CSF) [72,73] and the tissue factor (TF) [74].

Assays described in this work, designed to test the effect of media conditioned by senescent cells on platelet aggregation, adhesion and activation, were performed using three independently generated conditioned media. Each one of these media was tested, in triplicate, on platelets obtained from three independent donors. Thus, our results clearly show that the three senescent conditioned media were able to induce the adhesion of platelets to collagen-coated surfaces above the level of adhesion induced by media conditioned by non-senescent, control, cells (Figure 3). The same senescent conditioned media, tested in platelets from at least three independent donors, were able to induce platelet aggregation under sub-aggregating concentrations of the agonist ADP. These data indicate that additional factors, not present in the SASP, are necessary to prime platelets for aggregation. Interestingly, these responses were similar to those reported in platelets derived from older adults, which were also tested under sub-aggregating concentrations of agonist [10]. Platelets purified from older adults responded to concentrations of agonist that normally should not induce aggregation. It is tempting to speculate that an increase in the pool of senescent cells in these individuals could explain the basal platelet activation state observed.

Taken together, our results suggest that the secretory phenotype of senescent endothelial cells can play a crucial role in modulating platelet function, stimulating platelet activation, adhesion and aggregation. This, in turn, could partly explain age-dependent platelet alterations and atherothrombotic events.

## 4. Materials and Methods

### 4.1. Cell Culture

Human microvascular endothelial cells (HMEC-1) were kindly provided by Dr. Fernando Delgado from Universidad Católica del Maule (UCM). HMEC-1 cells were propagated in MCDB-131 endothelial basal medium, supplemented with 10% fetal bovine serum (FBS), 10 ng/mL of epidermal growth factor (EGF), 1 µg/mL of hydrocortisone, 2 mM of L-glutamine, 100 U/mL of penicillin, 25 µg/mL of amphotericin B and 100 µg/mL of streptomycin. Cells were maintained at 37 °C in 5% CO_2_ in a humidified environment.

### 4.2. Doxorubicin-Induced Senescence

HMEC-1 cells were cultured for different times in the presence or absence of increasing concentrations of doxorubicin. Briefly, HMEC-1 cells were seeded on 6-well plates (5 × 10^4^ cells per well) and then grown for 48 or 96 h in the presence or absence of 0.05, 0.1 and 0.5 µM of doxorubicin. doxorubicin’s effects on proliferation (proliferation curves) and senescence (SA-β-galactosidase assay) were recorded and quantified. After selecting 0.05 µM as the minimal concentration of doxorubicin capable of inducing senescence without affecting cell viability, 2 × 10^4^ vehicle-treated control cells and 1 × 10^5^ doxorubicin-treated cells were plated and then treated with 0.05 µM doxorubicin for 24, 48, 72 and 96 h. The different numbers of cells initially seeded were justified by differences in their proliferative capacity in order to avoid overgrowth of control cells.

### 4.3. Senescence-Associated β-GALACTOSIDASE (SA-β-Gal) Assay

Induction of cellular senescence was confirmed by in-situ detection of SA-β-Gal activity. Briefly, adherent cells growing on coverslips were washed once with 1x PBS and then fixed in 2% formaldehyde/0.2% glutaraldehyde/1× PBS for 10 min at room temperature. After three additional washes with 1× PBS, cells were incubated in β-galactosidase staining solution (1 mg/mL 5-bromo-4-chloro-3-indolyl-β-d-galactoside (X-Gal); 40 mM citric acid/sodium phosphate, pH 6.0; 5 mM potassium ferrocyanide; 5 mM potassium ferricyanide; 150 mM NaCl; 2 mM MgCl_2_) at 37 °C, overnight. After this incubation, stained cells were washed with 1× PBS and mounted in glycerol. The images were acquired in an Olympus BX53 microscope (Olympus). Digital images were taken using Q-Capture Pro 7 software (QImagine, Surrey, BC, Canada).

### 4.4. Quantitative Real-Time PCR (qRT-PCR) Analyses

Total RNA was prepared from HMEC-1 cells using the TRIzol^®^ Reagent (Life Technologies™, Carlsband, CA, USA), essentially following the manufacturer’s protocol. Reverse transcription was carried out with the First Strand cDNA Synthesis kit (Thermo Fisher Scientific, Carlsband, CA, USA). For quantitative real-time PCR (qRT-PCR), Maxima SYBR Green/ROX qPCR Master Mix (2×) (Thermo Fisher Scientific, Carlsband, CA, USA) was used. The following cycling conditions were used: an initial cycle at 95 °C for 10 min, followed by 40 cycles at 95 °C for 15 s (sec), 60 °C for 15 sec, 72 °C for 20 sec, and a final cycle at 95 °C for 1 min. Reactions were run in a Stratagene Mx3000P real-time thermal cycler. The results were analyzed with the MxPro QPCR software (Agilent Technologies, Santa Clara, CA, USA) and relative expression of mRNAs was calculated using the 2^ΔΔ*C*t^ method. The ribosomal gene *RPL19* was used to normalize gene expression levels. All qRT-PCR primers are listed in Table S1.

### 4.5. Harvesting of Conditioned Media

Media in which non-senescent and senescent HMEC-1 cells were cultured (conditioned media) were collected for functional analyses. Briefly, 2 × 10^4^ and 1 × 10^5^ HMEC-1 cells were cultured for 72 h in the presence of vehicle (0.01% DMSO) or doxorubicin (0.05 µM; MP Biomedicals, LLC, Santa Ana, CA, USA), respectively. Following this incubation time, media were replaced with minimum volumes of serum- and doxorubicin-free media, and cells were cultured for an additional 24 or 48 h. Conditioned media were collected and centrifuged for 5 min at 5000× *g* (D3024R microcentrifuge, SCILOGEX, EEUU) before use. Finally, protein concentrations were estimated by Bradford assays using a BSA-based calibration curve.

### 4.6. Determination of IL-1β in Conditioned Media

In order to quantify interleukin-1β (IL-1β) in media conditioned by senescent and non-senescent HMEC-1 cells, an enzyme-linked immunosorbent assay (ELISA) was utilized (Cat. No. BMS224HS; eBioscience, San Diego, CA, USA). Briefly, 50 μL of serum- and doxorubicin-free conditioned medium, collected 24–48 h after a 72-h period of senescence induction, were added to wells containing immobilized anti-IL-1β antibodies. Biotin–streptavidin complexes and colorimetric reagents were added for signal amplification. Finally, signals were detected in a Synergy HTX Multi-Mode Reader (Biotek instrument, Winooski, VT, USA) at 450 nm. The results shown are mean ± SD from three separate samples.

### 4.7. Platelet-Enriched Plasma (PRP)

Healthy volunteers were subjected to venous blood withdrawal after signing an informed consent document. Before the procedure, a short survey was applied in order to ensure that all individuals met inclusion criteria. Both the informed consent and the survey were previously approved by the Scientific Ethics Committee of the University of Talca (code number 2015-102-NB). The following were inclusion criteria used to select blood donors: male gender, age between 25 and 30 years, absence of chronic or infectious diseases, absence of hematological or coagulation diseases, body mass index (BMI) values between 18.5 and 24.9, cessation of consumption of nonsteroidal anti-inflammatory drugs at least 10 days before blood withdrawal, and no alcohol and tobacco consumption during the previous 10 days before blood withdrawal. Blood samples were collected in tubes containing 3.2% of the anticoagulant sodium citrate, in a 9:1 ratio (blood: anticoagulant). Platelet-rich plasma (PRP) was obtained by centrifugation at 240× *g* for 10 min at room temperature (RT). Two-thirds of PRP were removed. The original tubes were centrifuged at 160× *g* for 10 min to obtain the platelet-poor plasma (PPP). Finally, the PRP was adjusted to 200,000 platelets/μL with PPP. The number of platelets was quantified using an automated hematology analyzer (BC-2800 Mindray Analyzer, Shenzhen, China).

### 4.8. Washed Platelets

For the preparation of washed platelets, PRP was combined with calcein-AM (Santa Cruz Biotechnology) to a final concentration of 4 μM. The mixture was further incubated for 1 h at 37 °C in the dark and under constant stirring. The PRP was then centrifuged at 3700 RPM for 5 min at room temperature. The pellet was washed with HEPES-Tyrode buffer (120 nmol/L of PGE1, pH 6.2) followed by an additional centrifugation at 700 RPM for 5 min and at room temperature. Finally, washed platelets were resuspended in serum-free culture medium (MCDB131) to a concentration 200,000 platelets/μL. The number of platelets was quantified using an automated hematology analyzer (BC-2800 Mindray Analyzer, Shenzhen, China).

### 4.9. Platelet Adhesion Assays

Platelet adhesion assays, in the presence or absence of media conditioned by senescent cells, were performed under static conditions following a modified version of the method described by Stevens [75]. Briefly, collagen (20 μg/mL) was first added to each well of a 96-well plate and let to gelify for 1 h at 37 °C. Any remaining coating solution was then removed, and the wells were washed twice with 1× PBS. After removing the PBS, the wells were filled completely with 2% (*w/v*) bovine serum albumin (BSA) diluted in 1× PBS and further incubated for 1 h at 37 °C. Finally, the wells were washed twice in 1× PBS and air-dried before using them in adhesion assays.

For platelet adhesion assays, 50 μL of washed platelets (1 × 10^7^ platelets/well) were combined with 50 μL of medium conditioned by senescent or non-senescent HMEC-1 cells. The mixture was then added to collagen-coated wells in 96-well plates and incubated for 1 h at 37 °C, under dark conditions. Adhered platelets were then fixed in 4% (*w/v*) paraformaldehyde (PFA), pH 7.4, at room temperature for 15 min. Total fluorescence per well was measured in a Fluorometer (Synergy HTX Multi-Mode Microplate Reader; Biotek Instruments, Winooski, VT, USA) equipped with filters that provide wavelength selection (excitation at 494 nm; emission at 517 nm). Plates were extensively washed with 1× PBS in order to remove loosely adherent platelets. A second read was then performed to capture the remaining fluorescence, which represented strongly adhered platelets. The proportion of adherent platelets was calculated as a proportion of the total fluorescence in an individual well before washing. Samples from each volunteer were independently processed for each assay.

### 4.10. Platelet Aggregation Assays

For platelet aggregation assays, 450 µL of PRP was incubated with 50 µL of saline serum (NaCl 0.9%), 50 µL of non-senescent endothelial cell conditioned medium or 50 µL of senescent endothelial cell conditioned medium, for 5 min at 37 °C. A quantity of 10 µL of the platelet aggregation agonist ADP (2 µM) was then added to the samples, and platelet aggregation was recorded for 6 min. The results of platelet aggregation were determined by the software AGGRO/LINK (Chrono-Log, Havertown, PA, USA). 

### 4.11. Flow Cytometry-Based Analyses for P-Selectin and Activated Glycoprotein (GP)IIb/IIIa

Platelet activation in response to media conditioned by senescent cells was determined by flow cytometry. To this end, an FITC-labeled antibody that recognizes the active conformation of the αIIbβ3 receptor (PAC-1) and a PE-labeled anti-CD62 antibody that binds to exposed P-selectin on the surface of platelets were used (BD Biosciences (San Jose, CA, USA). The presence of these markers on platelets was detected in an Accuri C6 Flow Cytometer (BD, Biosciences). Briefly, 450 µL of PRP (2 × 10^5^ platelets/µL) were combined with 50 µL media conditioned by senescent or non-senescent HMEC-1 cells, and the mixture was incubated for 5 min at 37 °C, in the presence or absence of sub-aggregating concentrations of ADP (2.0 μM). A quantity of 50 µL of the sample was then combined with saturating amounts of anti-PAC-1 and anti-CD62-PE antibodies and incubated for 30 min at room temperature in the dark before being analyzed by flow cytometry. Platelet populations were gated based on cell size using CD61, as well as forward and side scatter parameters. Over 10,000 events were analyzed in each case. All analyses were performed with the BD Accuri C6 Software (BD Biosciences). Fold change was calculated by dividing the median fluorescence intensity (MFI) of platelets exposed to media conditioned by senescent cells by the MFI of platelets exposed to media conditioned by non-senescent cells.

### 4.12. Statistical Analyses

Data were compiled and analyzed with the SigmaPlot software package, version 12.0 (Systal Sofware, Chicago, IL, USA). Group differences were calculated with t-student. Differences with *p*-values < 0.05 were considered statistically significant, and all data were shown as mean ± standard error of the mean (SD).

## Supplementary Materials

The following are available online at https://www.mdpi.com/1422-0067/21/9/3287/s1.
