# Supplementary material for: Platelet Activation Is Triggered by Factors Secreted by Senescent Endothelial HMEC-1 Cells In Vitro
Source: Int J Mol Sci. 2020 May 6;21(9):3287. doi: 10.3390/ijms21093287 (PMC7246568; doi:10.3390/ijms21093287)
Supplement: Supplementary file 1 [file ijms-21-03287-s001.docx]

Table S1. **Primer sets used for quantitative real time PCR analyses.**

| **Gene** | **GeneBank** | **Forward** | **Reverse** |
| --- | --- | --- | --- |
| *IL-6* | NM_000600.5 | FGGCACCTCAGATTGTTGTTGTT | GTGTCCTAACGCTCATACTTTTAGT |
| *IL-8* | NM_000584.4 | AGGCACAAACTTTCAGAGACAGCAG | TGTTTACACACAGTGAGATGGTTCC |
| *IL-1α* | NM_000575.4 | AGATGCCTGAGATACCCAAAACC | CCAAGCACACCCAGTAGTCT |
| *IL-1β* | NM_000576.2 | ATGATGGCTTATTACAGTGGCAA | GTCGGAGATTCGTAGCTGGA |
| *G-CSF* | NM_000759.3 | CACGAGGGTCAGGACTGTGACTCTT | ACATGATTCCTCCTGTCTGCTCCC |
| *GM-CSF* | NM_000758.3 | TTCTGCTTGTCATCCCCTTT | TGCCTGTATCAGGGTCAGTG |
| *PDGFA* | NM_002607.5 | AGAACTATGCGTCAACCAATCG | ACACCAACAACACAGACAGAAGC |
| *PDGFB* | NM_002608.4 | TAGAGATGGAGTTTGCTGTTGAGG | AGAGAGATGAAAGGAACCAGAGGA |
| *TF* | NM_001063.4 | GAACCCAACAACAAAGAGGGATAC | TACCATCAAGGCACAGCAACTC |
| *MIF* | NM_002425.2 | AGTGGTGTCCGAGAAGTCAGGCA | TTGCTGTAGGAGCGGTTCTGCG |
| *Gro-Alfa* | NM_001511.4 | TGAGGAGCCTGCAACATGCCA | GCCCCTTTGTTCTAAGCCAGAAACA |
| *CDKN1A* | NM_000389.5 | TGTCCGTCAGAACCCATGC | AAAGTCGAAGTTCCATCGCTC |
| *RPL19* | NM_000981 | CATCCGCAAGCCTGTGACG | TGTGACCTTCTCTGGCATTCG |
